# Longitudinal Linkages Between Parent-Child Discrepancies in Reports on Parental Autonomy Support and Informants’ Depressive Symptoms

**DOI:** 10.1007/s10964-022-01733-y

**Published:** 2023-01-24

**Authors:** Paula Vrolijk, Caspar J. Van Lissa, Susan Branje, Renske Keizer

**Affiliations:** 1 Department of Public Administration and Sociology, Erasmus University Rotterdam, P.O. Box 1738, 3000 DR Rotterdam, The Netherlands; 2 Department of Methodology and Statistics, Tilburg University, Tilburg, Netherlands; 3 Department of Youth and Family, Utrecht University, Utrecht, Netherlands

**Keywords:** Parent-child discrepancies, Adolescence, Autonomy support, Depressive symptoms, Longitudinal research

## Abstract

Although parent-child discrepancies in reports of parenting are known to be associated with child depressive symptoms, the direction of causality is unknown. To address this knowledge gap, this study contributes to existing literature by examining longitudinal within-family linkages between parent-child discrepancies in their reports on autonomy support and depressive symptoms of children, while also assessing these linkages with parents’ depressive symptoms. In addition, this study explored whether these linkages differ for father- versus mother-child discrepancies. Longitudinal data (six annual waves) of 497 adolescents (56.9% boys, *M*_*age*_ at T_1_ = 13.03, *SD* = 0.46), their mothers (*N* = 495), and their fathers (*N* = 446) of the Dutch study Research on Adolescent Development and Relationships (RADAR) were used. Counter to expectations, the results of a Random Intercept Cross-Lagged Panel Model provided no evidence for within-family cross-lagged effects. Instead, stable differences between families explained linkages; in families where children reported on average higher levels of depressive symptoms, children also reported lower levels of autonomy support relative to their parents. There were no associations between parent-child discrepancies and *parents’* depressive symptoms. Thus, the findings suggest that depressive symptoms are neither a consequence, nor a predictor of parent-child discrepancies in adolescence. The hypotheses and analytical plan of this study were preregistered in a project on the Open Science Framework.

## Introduction

Parents and children have their own unique views on parenting (de Los Reyes & Ohannessian, 2016) which influence how they report on the parent-child relationship (e.g., Van Lissa et al., 2015). Studying these discrepancies is important, as they are known to be related to child maladjustment, such as depressive symptoms (e.g., Human et al., 2016). Parent-child discrepancies in reports on parenting might be a risk factor for developing depressive symptoms, yet only one study truly examined the direction of effects (Nelemans et al., 2022). It could also be the case that depressive symptoms contribute to reporter discrepancies, as they may fuel negatively biased perspectives (Richters, 1992). Obtaining a better understanding of the direction of effects between these constructs has clinical relevance since it provides information about whether parent-child discrepancies are, indeed, a potential risk factor. This study aimed to examine the direction of effects by studying whether, at the within-family level, changes in parent-child discrepancies predict changes in depressive symptoms, and vice versa. This study also extended previous research by considering both children’s *and* parents’ depressive symptoms. Finally, differences between father- and mother-child discrepancies were explored.

### Theoretical Explanations for Parent-Child Discrepancies in Autonomy Support

According to Self-Determination Theory (SDT), autonomy-supportive parents accept their children’s opinions, take their perspectives, and encourage them to act on their own interests and values (Deci & Ryan, 2000). In adolescence, researchers often rely on either child or parent reports to measure parental autonomy support (McCurdy et al., 2020). Parents’ and children’s perspectives on autonomy support may differ because parents’ view of their own parenting is often more favorable than those of their children (Hou et al., 2020). The *generational stake hypothesis* implies that parents and children consistently view the same interactions and behaviors from different perspectives, because children have a stake in maximizing autonomy, whereas parents have a stake in socializing their children (Bengtson & Kuypers, 1971). Although parents may feel that they are autonomy-supportive, the *stage-environment fit theory* states that adolescents’ search for independence and self-identity could make their expectations of parental autonomy support different from what parents are able to provide (Eccles et al., 1993). Empirical research indeed showed that, on average, adolescents report lower autonomy support relative to their parents (Sher-Censor et al., 2011; Vrolijk et al., 2020), and less positive relationships with parents in general (Van Lissa et al., 2015).

### Bidirectional Linkages Between Parent-Child Discrepancies and Depressive Symptoms

Although discrepancies in reports on autonomy support during adolescence are normative, individual differences in these discrepancies might relate to adolescent and parent depressive symptoms. Previous research found that larger discrepancies in reports on autonomy support were associated with more depressive symptoms and lower self-worth in adolescents (Sher-Censor et al., 2011). Cross-sectional studies on associations between parent-child discrepancies in parenting and *parents’* depressive symptoms showed mixed results: Whereas some found no association between parents’ depressive symptoms and parent-child discrepancies (Korelitz, 2017; Ohannessian et al., 2016), others showed that mothers with more depressive symptoms report less positive (relative to children) about their parenting (Pérez et al., 2018; Shishido & Latzman, 2017). One study found that both mothers’ and adolescents’ depressive symptoms were associated with perceiving more conflict relative to the other respondent (Ehrlich et al., 2016).

Linkages between parent-child discrepancies and informants’ depressive symptoms could signal that discrepancies cause future depressive symptoms. According to the *Operations Triad Model*, parent-child discrepancies are a risk for developing internalizing problems, because they indicate low awareness and misunderstanding within the parent-child relationship (de Los Reyes & Ohannessian, 2016). A longitudinal study revealed that when adolescents had more negative views on family functioning relative to parents, adolescents reported more future depressive symptoms (Human et al., 2016). Parent-child discrepancies might also predict parents’ future depressive symptoms. Research found that autonomy-supportive parenting predicted parental need fulfillment over time (Neubauer et al., 2021). This may indicate that difficulties in the parent-child relationship can also influence parental adjustment.

The association between parent-child discrepancies and informants’ depressive symptoms might also reflect an effect of depressive symptoms on future parent-child discrepancies. The *depression-distortion hypothesis* (Richters, 1992) posits that individuals with depressive symptoms disproportionately remember negative information, which affects their perspectives on social interactions. When adolescents had more difficulties than usual with regulating their emotions, this predicted lower levels of child-perceived maternal autonomy support over time (Keskin & Branje, 2022). A recent longitudinal study on the same cohort as this study provided more insight in the direction of effects between discrepancies in mother- and child-reported conflict and warmth and child internalizing problems (Nelemans et al., 2022). In support of the *depression-distortion hypothesis*, these results showed that depressive symptoms predict parent-child discrepancies more strongly than vice versa, but only for conflict and not for warmth. Specifically, greater adolescent-reported depressive symptoms predicted a greater increase in adolescent-reported conflict than mother-reported conflict. At present, bidirectional linkages between *parent* depressive symptoms and parent-child discrepancies have not been investigated longitudinally.

Previous research was mostly cross-sectional, thus could not address the direction of effects between depressive symptoms and parent-child discrepancies. In addition, the few longitudinal studies used a between-family design. To gain a deeper understanding of the direction of effects, it is important to examine whether within-family fluctuations from the usual level of parent-child discrepancies, predict within-family changes in informants’ depressive symptoms one time-point later or vice versa (see Hamaker et al., 2015).

### Differences Between Father- and Mother-Child Discrepancies

Father-child and mother-child discrepancies might be differentially related to depressive symptoms. Theory suggests that differences between father-child and mother-child discrepancies might be primarily due to differences in parents’ reports. According to the *perceiver effect*, there are individual differences in people’s judgment of relationships (Kenny & la Voie, 1984). Adolescents display a stronger perceiver effect and tend to rate relationship quality with fathers’ and mothers’ similarly (Branje et al., 2002). However, children might also interpret fathers’ and mothers’ behavior differently because of different experiences and expectations (Palkovitz et al., 2014). Although fathers’ and mothers’ roles are becoming increasingly similar (Fagan et al., 2014), mothers still invest more time in childrearing and experience stricter societal expectations about adherence to the maternal role (Parke & Cookston, 2019). Because mothers are more involved, adolescents may experience more conflicts about daily hassles with their mothers compared to their fathers (e.g., Branje et al., 2012) resulting in lower levels of child-perceived maternal autonomy support. Parents’ self-reports are particularly likely to be impacted by socially sanctioned roles and desirability bias. Mothers may therefore be more inclined to answer questions about parenting in a socially desirable way in line with their role, which could enlarge mother-child discrepancies. Empirical research indeed showed that parent-child discrepancies were larger for mothers versus fathers (e.g., Ingoglia et al., 2021). In addition, father-child discrepancies in levels of conflicts were more strongly related to adolescent depressive symptoms compared to mother-child discrepancies (Nelemans et al., 2016; Sher-Censor et al., 2011). However, none of these prior studies examined effects at the within-family level. It thus remains to be seen whether these findings translate to within-family associations.

Till date, studies did not compare father- and mother-child discrepancies in relation to parents’ depressive symptoms. When the pressure of being a ‘good parent’ is higher for mothers compared to fathers, increases in mother-child discrepancies over time may arise because of this pressure and these difficulties in the parent-child relationship may have a larger effect on mothers’ depressive symptoms than on fathers’ depressive symptoms.

## Current Study

Although there is ample evidence for an association between parent-child discrepancies in perceptions of parenting and depressive symptoms, little is known about the direction of effects. To address this knowledge gap, the current study examined within-family longitudinal associations between parent-child discrepancies in perceived parental autonomy support and informants’ depressive symptoms. First, it was examined whether within-family changes in parents’ and children’s depressive symptoms predicted future within-family changes in parent-child discrepancies or vice versa. In accordance with the *depression-distortion hypothesis*, it was hypothesized that, within families, increased child depressive symptoms would predict a decrease in child-reported autonomy support relative to parent reports (Hypothesis 1a). It was further hypothesized that, within families, increased parent depressive symptoms would predict a decrease in parent-reported autonomy support relative to child reports (Hypothesis 1b). Furthermore, it was hypothesized that, within families, decreases in child reports of autonomy support relative to parent reports would predict increased child- (Hypothesis 1c) and parent depressive symptoms (Hypothesis 1d). Finally, this study explored whether these longitudinal associations between depressive symptoms and parent-child discrepancies differed for fathers and mothers. It was hypothesized that parent-child discrepancies would predict depressive symptoms more strongly for mothers than fathers (Hypothesis 2).

## Method

### Participants

Data were drawn from the first six annual measurement waves of the ongoing longitudinal study Research on Adolescent Development And Relationships (RADAR)-young (Branje & Meeus, 2018) consisting of questionnaires from 497 adolescents (56.9% boys, *M*_age_ at T_1_ = 13.03, *SD* = 0.46), their mothers (*N* = 495, *M*_age_ at T_1_ = 44.41, *SD* = 4.45), and their fathers (*N* = 446, *M*_age_ at T_1_ = 46.74, *SD* = 5.10). Most of the adolescents were of Dutch origin (95%), came from families classified as having a medium or high socioeconomic status (90%), lived with both parents (86%), and answered questions about their biological mother (99%) and biological father (89%). Sample size determination, data exclusions, and all manipulations of measures are reported below.

Approximately 13% of the families dropped out between the first and sixth wave. The majority of the adolescents (86%), mothers (85%), and fathers (76%) still participated in the sixth wave. Jamshidan and Jalal’s non-parametric MCAR was non-significant (*p* = 0.095) indicating that there was no association between observed values and missingness. Missing data were imputed on scale level using the *missForest-*package in R such that all models were run on *N* = 497 (Stekhoven & Bühlmann, 2012). All analyses were conducted in R version 4.0.2 (R Core Team, 2020). All code, output, supplementary materials, and preregistration are available in a project on the Open Science Framework: https://osf.io/dz69y/. The RADAR-young dataset is archived in the DANS repository under the title Research on Adolescent Development And Relationships (RADAR; young cohort), and available by restricted access.

### Measurements

See Appendix A for complete psychometric analyses.

#### Autonomy support

Respondents completed the ‘balanced relatedness’ scale, consisting of seven questions tapping into the extent to which parents accept the opinions, wishes, and needs of the child (Shulman et al., 1997). Children reported on autonomy support perceived from their father and mother separately, and parents reported on their own autonomy supportive behavior. The four-point scale ranged from (1) *absolutely disagree* to (4) *absolutely agree*. Example items are: “My father/mother respects my decisions” or “I consider the opinion of my child”. After establishing measurement invariance, mean scores were computed for all respondents. Previous studies support construct validity, convergent validity, and test-retest reliability (Shulman et al., 1997; van der Giessen et al., 2013). In this sample, the scale had good reliability across respondents and waves, Cronbach’s α [0.79, 0.90].

#### Depressive symptoms adolescents

Adolescents completed the *Reynolds Adolescent Depression Scale 2*^*nd*^ *edition* (RADS-2; Reynolds, 2002), consisting of 23 items with a four-point scale ranging from (1) *almost never* to (4) *most of the time*. Example items are: “I am sad” or “I feel life is unfair”. Previous research showed good internal consistency, test-retest reliability, and validity (Reynolds, 2002). In the present study, a scale composed of mean scores had good reliability on each wave, Cronbach’s α [0.93, 0.95].

#### Depressive symptoms parents

Parents completed the depression subscale of the *Adult Self Report* of the Achenbach System of Empirically Based Assessment (Achenbach & Rescorla, 2003). This scale consists of 18 items assessing parents’ depressive symptoms in the past six months measured on a three-point scale, ranging from (0) *not true* to (2) *very true or often true*. Example items are: “I am unhappy, sad, or depressed” or “I feel like I can’t succeed”. Previous research provide evidence for good validity and reliability (Rescorla & Achenbach, 2004), and the current study showed good reliability for mean scale scores for both respondents across waves, Cronbach’s α [0.81, 0.91].

### Analysis

#### Measurement invariance

For longitudinal research, it is important to ascertain that constructs carry the same meaning from one year to the next (McCurdy et al., 2020). Furthermore, establishing *measurement invariance* across respondents is important for meaningful interpretation of reporter discrepancies (Russell et al., 2016). To this end, measurement invariance was established using semTools (Jorgensen et al., 2021). Three Confirmatory Factor Analysis (CFA) models were compared to test configural invariance, metric invariance, and scalar invariance (see Little, 2013) across respondents and time points. In this process, response categories ‘completely disagree’ and ‘disagree’ were merged for all items to address problems in model convergence caused by too few responses in these categories (Liu et al., 2017). Model fit was assessed using the Root-Mean-Square Error of Approximation (RMSEA; below 0.05 indicates good model fit), Standardized Root Mean-Square Residual (SRMR; below 0.08 indicates good model fit) and Comparative Fit Index (CFI) and Tucker-Lewis Index (TLI; above 0.90 indicates adequate model fit; above 0.95 indicates good model fit). Invariance constraints resulted in negligible changes in model fit, indicating that the assumption of measurement invariance was supported (CFI change ≤ −0.010, RMSEA change ≤ 0.015, SRMR change ≤ 0.030 (Chen, 2007). Although the Satorra-Bentler tests were significant, results showed that all changes in CFI, RMSEA and SRMR were below the specified cutoff criteria (see Table 1). Since chi-square difference tests are affected by complexity of the model and sample size, Satorra-Bentler difference tests should not be used as a sole indicator of model fit (Cheung & Rensvold, 2002). Taken together the results of all fit indices, longitudinal measurement invariance across groups was assumed.

**Table 1:** Model Fits of Models Testing Measurement Invariance

Model	χ^2^	DF	SRMR	RMSEA	CFI	TLI	Δ χ^2^ *p*
1. Configural	4830.14	2796	0.052	0.038	0.997	0.966	
2. Metric	5246.67	2934	0.052	0.040	0.996	0.996	<0.001
3. Scalar	5863.10	3072	0.052	0.043	0.996	0.995	<0.001

*SRMR* standardized root mean-square residual, *RMSEA* root-mean-square error of approximation, *CFI* comparative fit index, *TLI* Tucker-Lewis index, Δ χ^2^ *p* = Satorra-Bentler Scaled Chi-Square difference

#### Analysis strategy

**Fig. 1:**
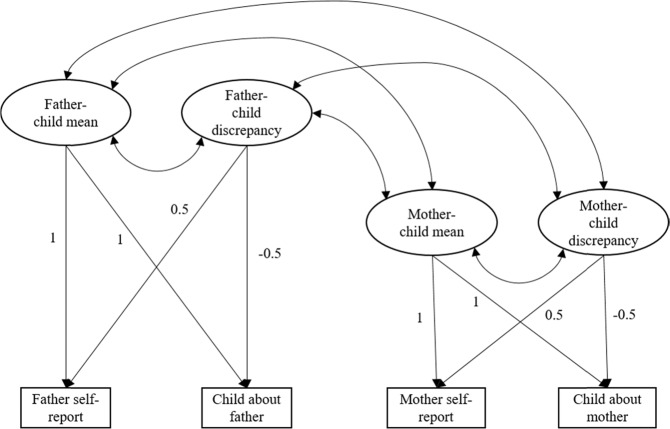
Latent Congruence Model. Note. Estimation of latent mean and difference. These latent variables were estimated on the within-family level, for each wave separately. The residuals of the observed variables were fixed to 0

Parents’ and children’s observed autonomy support at each wave was re-expressed using two latent variables: one representing mean autonomy support, and one representing parent-child discrepancies (Cheung, 2009). These latent variables were allowed to covary, and the residuals of the observed variables were fixed to 0 such that their variance is entirely re-expressed as a latent mean and difference (see Fig. 1). This was done separately for fathers and mothers. To estimate parent-child discrepancies, factor loadings of parent-reported autonomy support were fixed at 0.5 and factor loadings of child-reported autonomy support were fixed at −0.5. Positive values of the latent discrepancies variables indicate that parents report higher levels of autonomy support relative to the child, and negative values mean that children report higher levels of autonomy support relative to the parent.

To answer the research questions, a Random Intercept Cross-Lagged Panel Model (RI-CLPM) was estimated which disentangles within-family from between-family associations (Hamaker et al., 2015; see Appendix B for the representation of a RI-CLPM). All hypotheses were examined within the same model. This way analyses were controlled for the effect of the other informant’s depressive symptoms as depressive symptoms of father, mother, and child may be related. As child depressive symptoms negatively influence child perspectives and parent depressive symptoms negatively influence parents’ perspective, they have a reversed effect on parent-child discrepancies. It is therefore important to include both child *and* parent depressive symptoms to control for each other’s influence and examine their individual contributions. Next, also the average perspectives on parenting were included, because these may be predicted by (Nelemans et al., 2022), and predictive of, depressive symptoms (Human et al., 2016).

**Fig. 2:**
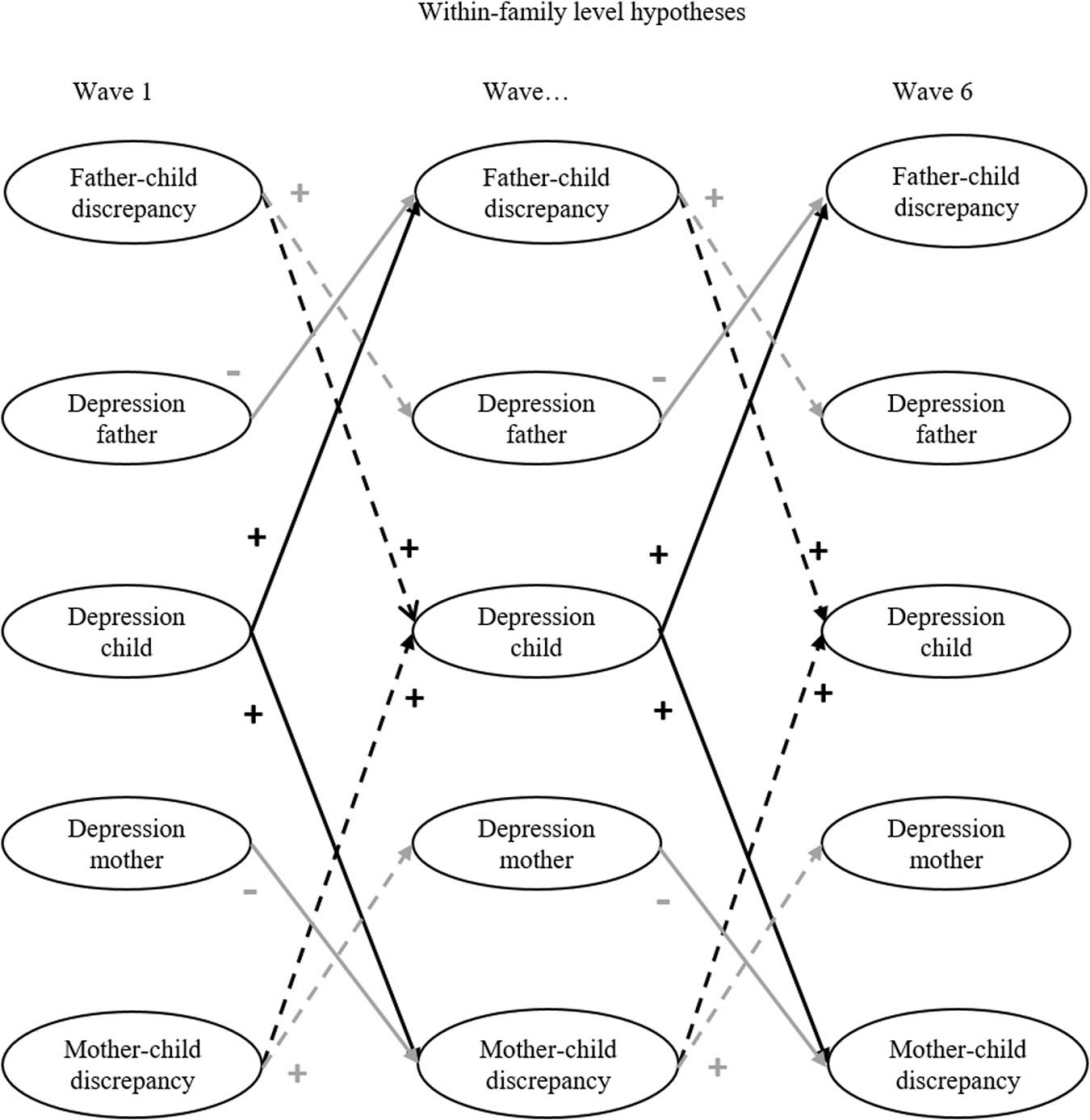
Within-Family Level Hypotheses. Note. Simple representation of the hypotheses on the within-family level. All between- and within-family associations between the variables were modeled (e.g., concurrent correlations and stability pathways). H1a = solid black, H1b = solid grey, H1c = dotted black, H1d = dotted grey

Intraclass Correlation Coefficients (ICC’s) showed that more than a third of the variance of father-child discrepancies (40%) and mother-child discrepancies (35%) in autonomy support was due to differences at the between-family level. Most of the variance of depressive symptoms of adolescents (61%), fathers (72%), and mothers (71%) was due to differences at the between-family level. The remainder of the variance is due to within-family fluctuations or residual (error) variance. It is therefore relevant to take into account between-family differences when examining the longitudinal associations between parent-child discrepancies in autonomy support and informants’ depressive symptoms. For reasons of parsimony, all within-time correlations, stability pathways, and cross-lagged coefficients were constrained over time. Model fit indices showed that these constraints were defensible; RMSEA, TLI, and CFI were comparable, and BIC became lower, indicating that the constrained model was preferred. The fit of the final constrained RICLPM was good, and this model was used to test the hypotheses (see Table 2 for the model building process and model fits). The research questions tap into associations on the within-family level (see Fig. 2 for a graphical representation of the hypotheses). Wald tests with Bonferroni-Holm correction were used to explore differences between fathers and mothers in associations between parent-child discrepancies and informants’ depressive symptoms. None of these Wald-tests were significant after correction.

**Table 2:** Model Fit Indices

Model	χ^2^	DF	scf	AIC	BIC	RMSEA	CFI	TLI	Δ χ^2^ *p*
1. CLPM	2055.05	493	1.14	3182.71	5084.99	0.09	0.86	0.76	
2. RI-CLPM	644.28	465	1.06	1593.97	3614.09	0.03	0.98	0.97	<0.001
3. Constrained RI-CLPM	976.22	735	1.15	1489.49	2373.29	0.03	0.98	0.97	0.003

*AIC* Akaike information criterion, *BIC* Bayesian information criterion, *RMSEA* root-mean-square error of approximation, *CFI* comparative fit index, *TLI* Tucker-Lewis index, Δ χ^2^ *p* = Satorra-Bentler Scaled Chi-Square difference, *Constrained RI-CLPM* Random Intercept Cross-Lagged Panel Model with constrained associations over time

## Results

### Descriptives

Descriptive statistics are shown in Table 3. Concurrent associations between these variables in the first wave are presented in Table 4, and subsequent waves are presented in the Appendix C. Concurrent associations of between child-reported with father-reported (*r* = 0.13 to *r* = 0.26, *p* < 0.05) and mother-reported autonomy support (*r* = 0.12 to *r* = 0.22, *p* < 0.05) were small to moderate.

**Table 3:** Means and Standard Deviations of Observed Variables

	Wave 1	Wave 2	Wave 3	Wave 4	Wave 5	Wave 6
	*M (SD)*	*M (SD)*	*M (SD)*	*M (SD)*	*M (SD)*	*M (SD)*
Father autonomy support						
Child-reported	3.25 (0.40)	3.20 (0.46)	3.18 (0.46)	3.17 (0.44)	3.14 (0.46)	3.12 (0.50)
Father-reported	3.25 (0.36)	3.26 (0.35)	3.25 (0.34)	3.25 (0.34)	3.29 (0.35)	3.28 (0.37)
Father-child discrepancies	−0.01 (0.50)	0.06 (0.52)	0.07 (0.48)	0.08 (0.50)	0.13 (0.50)	0.16 (0.55)
Mother autonomy support						
Child-reported	3.27 (0.41)	3.24 (0.44)	3.21 (0.44)	3.20 (0.43)	3.19 (0.44)	3.18 (0.45)
Mother-reported	3.27 (0.35)	3.31 (0.36)	3.31 (0.35)	3.35 (0.39)	3.38 (0.38)	3.43 (0.40)
Mother-child discrepancies	0.01 (0.49)	0.06 (0.50)	0.10 (0.52)	0.15 (0.53)	0.19 (0.55)	0.25 (0.53)
Depressive symptoms						
Child-reported	1.63 (0.49)	1.50 (0.50)	1.53 (0.52)	1.56 (0.54)	1.54 (0.51)	1.59 (0.55)
Father-reported	0.19 (0.18)	0.19 (0.22)	0.21 (0.23)	0.19 (0.22)	0.20 (0.25)	0.20 (0.25)
Mother-reported	0.25 (0.27)	0.23 (0.24)	0.22 (0.25)	0.22 (0.24)	0.22 (0.24)	0.22 (0.24)

**Table 4:** Concurrent Correlations Between Observed Variables During Wave 1

	1	2	3	4	5	6
1. Child-reported father support						
2. Child-reported mother support	0.64***					
3. Father-reported support	0.13**	0.01				
4. Mother-reported support	0.21***	0.18***	0.19***			
5. Child depressive symptoms	−0.34***	−0.23***	−0.12*	−0.11*		
6. Father depressive symptoms	−0.03	0.00	0.06	0.04	0.06	
7. Mother depressive symptoms	−0.09*	−0.07	−0.04	−0.03	0.21***	0.09

*p < 0.05; **p < 0.001

The final RI-CLPM provides more descriptive information about the differences in reports between parents and children (see Table 5). The mean of the random intercepts of parent-child discrepancies was positive and significant for fathers’ and mothers’ autonomy support indicating that, as expected, parents reported on average higher levels of autonomy support compared to their children. Mother-child discrepancies were on average more positive compared to father-child discrepancies, and this difference in intercepts was significant (Δ *β* = 0.16, *p* = 0.001). So, compared to father reports, mother reports on autonomy support were relatively more positive than child reports.

**Table 5:** Estimates Final Model

	Father autonomy support and symptoms			Mother autonomy support and symptoms			Difference F-M
	*B (SE)*	*p*	*β*	*B (SE)*	*p*	*β*	*p*
Between-family level							
Intercepts							
Intercept parent-child discrepancies	**0.07 (0.01)**	**<0.001**	**0.24**	**0.12 (0.01)**	**<0.001**	**0.40**	**0.001**
Intercept parent-child mean	**2.75 (0.01)**	**<0.001**	**12.56**	**2.81 (0.01)**	**<0.001**	**13.00**	**<0.001**
Associations random intercepts							
Discrepancies ↔ Symptoms child	**0.03 (0.01)**	**<0.001**	**0.29**	**0.03 (0.01)**	**<0.001**	**0.27**	0.966
Discrepancies ↔ Symptoms parent	0.00 (0.00)	0.915	0.01	0.01 (0.00)	0.100	0.09	0.230
Discrepancies ↔ Symptoms OP	0.01 (0.00)	0.110	0.09	−0.00 (0.00)	0.750	−0.02	0.153
Mean ↔ Symptoms child	**−0.03 (0.01)**	**<0.001**	**−0.40**	**−0.03 (0.01)**	**<0.001**	**−0.30**	0.025
Mean ↔ Symptoms parent	0.00 (0.00)	0.719	0.02	**−0.01 (0.00)**	**0.023**	**−0.12**	0.042
Mean ↔ Symptoms OP	**−0.01 (0.00)**	**0.014**	**−0.12**	0.00 (0.00)	0.252	0.06	**<0.001**
Symptoms parent ↔ Symptoms child	**0.01 (0.00)**	**0.035**	**0.12**	**0.02 (0.00)**	**<0.001**	**0.29**	**0.001**
Discrepancies ↔ Mean	−0.00 (0.00)	0.319	−0.07	0.01 (0.00)	0.180	0.09	0.018
Discrepancies ↔ Mean OP	**−0.02 (0.00)**	**<0.001**	**−0.35**	**−0.01 (0.00)**	**0.006**	**−0.20**	0.072
Discrepancies ↔ Discrepancies OP	**0.03 (0.01)**	**<0.001**	**0.41**				
Mean ↔ Mean OP	**0.03 (0.00)**	**<0.001**	**0.65**				
Symptoms parent ↔ Symptoms OP	**0.01 (0.00)**	**<0.001**	**0.30**				
Within-family level							
Concurrent associations Wave 1							
Discrepancies W1 ↔ Symptoms child W1	**0.02 (0.01)**	**<0.001**	**0.16**	0.01 (0.01)	0.302	0.05	0.110
Discrepancies W1 ↔ Symptoms parent W1	0.00 (0.00)	0.624	0.03	−0.01 (0.00)	0.294	−0.06	0.241
Discrepancies W1 ↔ Symptoms OP W1	0.00 (0.00)	0.708	0.02	0.00 (0.00)	0.726	0.02	0.891
Mean W1 ↔ Symptoms child W1	**−0.02 (0.00)**	**<0.001**	**−0.21**	**−0.01 (0.00)**	**0.006**	**−0.15**	0.513
Mean W1 ↔ Symptoms parent W1	0.00 (0.00)	0.731	0.02	−0.00 (0.00)	0.279	−0.06	0.258
Mean W1 ↔ Symptoms OP W1	−0.00 (0.00)	0.513	−0.03	0.00 (0.00)	0.326	0.06	0.236
Symptoms parent W1 ↔ Symptoms child W1	−0.00 (0.00)	0.627	−0.03	0.01 (0.01)	0.094	0.11	0.086
Discrepancies W1 ↔ Mean W1	**−0.02 (0.01)**	**<0.001**	**−0.26**	**−0.02 (0.01)**	**<0.001**	**−0.28**	0.482
Discrepancies W1 ↔ Mean OP W1	**−0.03 (0.01)**	**<0.001**	**−0.40**	**−0.02 (0.01)**	**<0.001**	**−0.27**	0.070
Discrepancies W1 ↔ Discrepancies OP W1	**0.06 (0.01)**	**<0.001**	**0.34**				
Mean W1 ↔ Mean OP W1	**0.01 (0.00)**	**<0.001**	**0.36**				
Symptoms parent W1 ↔ Symptoms OP W1	−0.00 (0.00)	0.796	−0.01				
Concurrent associations Wave 2 to Wave 6							
Discrepancies W2-6 ↔ Symptoms child W2-6	**0.01 (0.00)**	**0.002**	**0.08** – **0.11**	0.01 (0.00)	0.087	0.05 – 0.06	0.238
Discrepancies W2-6 ↔ Symptoms parent W2-6	−0.00 (0.00)	0.207	−0.03 – −0.04	−0.00 (0.00)	0.345	−0.02 – −0.03	0.853
Discrepancies W2-6 ↔ Symptoms OP W2-6	−0.00 (0.00)	0.790	−0.01 – −0.01	0.00 (0.00)	0.992	0.00 – 0.00	0.847
Mean W2-6 ↔ Symptoms child W2-6	**−0.01 (0.00)**	**<0.001**	**−0.10** – **−0.13**	**−0.01 (0.00)**	**0.002**	**−0.08** – **−0.11**	0.433
Mean W2-6 ↔ Symptoms parent W2-6	0.00 (0.00)	0.661	0.01 – 0.02	−0.00 (0.00)	0.352	−0.03 – −0.04	0.331
Mean W2-6 ↔ Symptoms OP W2-6	0.00 (0.00)	0.749	0.01 – 0.01	0.00 (0.00)	0.132	0.04 – 0.05	0.318
Symptoms parent W2-6 ↔ Symptoms child W2-6	0.00 (0.00)	0.154	0.04 – 0.05	**0.00 (0.00)**	**0.003**	**0.08 – 0.12**	0.167
Discrepancies W2-6 ↔ Mean W2-6	**−0.03 (0.00)**	**<0.001**	**−0.42** – **−0.51**	**−0.03 (0.00)**	**<0.001**	**−0.30** – **−0.35**	0.092
Discrepancies W2-6 ↔ Mean OP W2-6	**−0.02 (0.00)**	**<0.001**	**−0.24** – **−0.30**	**−0.02 (0.00)**	**<0.001**	**−0.30** – **−0.33**	0.121
Discrepancies W2-6 ↔ Discrepancies OP W2-6	**0.04 (0.01)**	**<0.001**	**0.27** – **0.30**				
Mean W2-6 ↔ Mean OP W2-6	**0.01 (0.00)**	**<0.001**	**0.31 – 0.38**				
Symptoms parent W2-6 ↔ Symptoms OP W2-6	0.00 (0.00)	0.100	0.05 – 0.06				
Cross-lagged effects							
Discrepancies → Symptoms child	0.02 (0.03)	0.525	0.02 – 0.02	0.02 (0.02)	0.341	0.02 – 0.03	0.918
Discrepancies → Symptoms parent	−0.01 (0.01)	0.463	−0.03 – −0.03	0.00 (0.01)	0.829	0.01 – 0.01	0.479
Discrepancies → Symptoms OP	0.00 (0.01)	0.788	0.01 – 0.01	0.00 (0.01)	0.618	0.01 – 0.02	0.892
Symptoms child → Discrepancies	0.01 (0.04)	0.757	0.01 – 0.01	0.01 (0.04)	0.865	0.01 – 0.01	0.914
Symptoms parent → Discrepancies	−0.04 (0.11)	0.725	−0.01 – −0.01	−0.08 (0.07)	0.277	−0.02 – −0.04	0.738
Symptoms parent → Discrepancies OP	−0.05 (0.09)	0.588	−0.01 – −0.02	−0.13 (0.07)	0.071	−0.03 – −0.04	0.610
Mean → Symptoms child	0.05 (0.05)	0.352	0.03 – 0.03	0.01 (0.05)	0.875	0.00 – 0.01	0.604
Mean → Symptoms parent	−0.00 (0.02)	0.962	−0.00 – −0.00	−0.02 (0.02)	0.343	−0.02 – −0.03	0.757
Mean → Symptoms OP	−0.00 (0.02)	0.865	−0.01– −0.01	0.03 (0.02)	0.116	0.04 – 0.05	0.833
Symptoms child → Mean	−0.01 (0.02)	0.791	−0.01 – −0.01	−0.02 (0.02)	0.406	−0.02 – −0.03	0.784
Symptoms parent → Mean	0.04 (0.05)	0.384	0.02 – 0.03	−0.06 (0.04)	0.080	−0.03 – −0.06	0.071
Symptoms parent → Mean OP	0.08 (0.05)	0.110	0.04 – 0.05	0.05 (0.04)	0.273	0.03 – 0.04	0.543
Symptoms parent → Symptoms child	0.04 (0.07)	0.508	0.01 – 0.02	0.09 (0.06)	0.122	0.03 – 0.05	0.605
Symptoms child → Symptoms parent	0.01 (0.01)	0.420	0.02 – 0.03	**0.03 (0.01)**	**0.010**	**0.07** – **0.08**	0.159
Discrepancies → Mean	**−0.04 (0.02)**	**0.024**	**−0.08** – **−0.09**	**−0.04 (0.02)**	**0.026**	**−0.08 – −0.09**	0.963
Mean → Discrepancies	−0.11 (0.07)	0.128	−0.05 – −0.06	−0.06 (0.07)	0.350	−0.03 – −0.03	0.602
Discrepancies → Mean OP	0.01 (0.02)	0.699	0.01 – 0.01	−0.02 (0.01)	0.105	−0.04 – −0.05	0.384
Mean OP → Discrepancies	0.03 (0.05)	0.586	0.01 – 0.02	**−0.16 (0.06)**	**0.012**	**−0.07 – −0.08**	0.016
Discrepancies → Discrepancies OP	0.01 (0.04)	0.756	0.01 – 0.01	0.01 (0.03)	0.662	0.01 – 0.01	0.993
Mean → Mean OP	0.03 (0.03)	0.365	0.03 – 0.03	−0.01 (0.03)	0.846	−0.01– −0.01	0.365
Symptoms parent → Symptoms OP	0.03 (0.03)	0.294	0.02 – 0.03	−0.02 (0.02)	0.347	−0.02 – −0.03	0.105

Boldface coefficients: *p* < 0.05, Difference F-M = Wald difference test between father and mother estimates, after Bonferonni-Holm correction none of the tests were significant, discrepancies = parent-child discrepancies in report on autonomy support, mean = average of parent and child report on autonomy support, symptoms = depressive symptoms, OP = other parent

Between-family level associations showed that, on average, in families where the parents and child reported higher mean levels of autonomy support, the child and mother reported fewer depressive symptoms. This was also true for mean levels of father autonomy support in relation to mothers’ depressive symptoms. Fathers’ depressive symptoms were unrelated to mean levels of autonomy support. Both father-child and mother-child discrepancies were positively related to child depressive symptoms on the between-family level. This means that in families where the difference between parent and child reports was larger, and parents reported more positively about their autonomy support than the child, children reported more depressive symptoms. The strength of this association did not differ significantly between father-child and mother-child discrepancies. There were no significant between-family associations between parent-child discrepancies and parents’ depressive symptoms. Further, the random intercept of father-child discrepancies was positively related to the random intercept of mother-child discrepancies. So, in families with higher levels of father-child discrepancies, in which father reports of autonomy support were more positive relative to child reports, there were also higher levels of mother-child discrepancies. Average levels of depressive symptoms of all respondents were positively correlated.

### Within-Family Associations Between Parent-Child Discrepancies and Depressive Symptoms

The estimates of the final model are presented in Table 5. According to Hypothesis 1, longitudinal bidirectional effects between parent-child discrepancies and informants’ depressive symptoms were expected. However, these cross-lagged effects were all non-significant, providing no evidence for Hypothesis 1. Hypothesis 2 was also not supported, as the effect of mother-child discrepancies on mother depressive symptoms was not stronger compared to the effect of father-child discrepancies on father depressive symptoms. These over-time effects were non-significant for both parents. There were also no differences between fathers and mothers in other within-family associations.

Only concurrent within-family associations between father-child discrepancies and informants’ depressive symptoms were significant: when father reports became more positive relative to child reports, children reported more depressive symptoms than usual. Although the concurrent within-family associations between parent-child discrepancies and child depressive symptoms were only significant for fathers’ autonomy support, and not for mothers’ autonomy support, Wald tests indicated that the covariance between discrepancies in autonomy support and depressive symptoms did not differ significantly between fathers and mothers in any of the waves (see Table 5).

On the within-family level, when mean levels of autonomy support were higher than usual during a time-point, this was related to decreased levels of *child* depressive symptoms but not to *parents’* depressive symptoms. In addition, there were no cross-lagged relations between mean levels of autonomy support and informants’ depressive symptoms.

### Sensitivity Analyses

Sensitivity analyses were conducted to examine whether substantive conclusions were the same when fathers and mothers were analyzed separately and when non-imputed data were used. These analyses did not lead to different conclusions about the hypotheses. Additionally, the data were analyzed with a CLPM without random intercepts. As this model had inadequate fit on all indices, its results could not be interpreted.

## Discussion

The present longitudinal study set out to gain a deeper understanding of the relations between parent-child discrepancies and depressive symptoms in adolescents and parents, examining over-time effects at the within-family level. This goes beyond prior research, which mostly used cross-sectional and between-family designs and thus could not speak to the direction of effects between these constructs. In addition, depressive symptoms of children *and* parents were considered, as well as father, mother, and child-reported autonomy support. The results showed that although larger discrepancies were associated to child depressive symptoms at the between-family level, parent-child discrepancies in autonomy support did not predict, nor were predicted by, informants’ depressive symptoms.

### Associations Between Parent-Child Discrepancies and Child Depressive Symptoms

The absence of over-time effects between child depressive symptoms and parent-child discrepancies provides no evidence for the *depression-distortion hypothesis* (Richters, 1992) or the *Operations Triad Model* (de Los Reyes & Ohannessian, 2016). Except for the concurrent within-family associations between child depressive symptoms and father-child discrepancies, associations between parent-child discrepancies and child depressive symptoms were mainly present on the between-family level, representing time-invariant, trait-like, differences between families. After taking into account mean perspectives on autonomy support, these between-family relations demonstrate that children who had a more negative view of autonomy support relative to their parents, also reported higher levels of depressive symptoms compared to other children. The results are in line with previous between-family research (Sher-Censor et al., 2011). A next step in research on parent-child discrepancies in autonomy support could be to examine stable individual or family characteristics which are related to higher levels of disagreement between parents and children, for instance, informers’ personality types (e.g., Mastrotheodoros et al., 2020) or parents’ personal norms about family obligations (e.g., Mandemakers & Dykstra, 2008). This will result in more information about the existence of parent-child discrepancies, and why some families are seen to have more parent-child discrepancies than others.

A possible explanation for not finding over-time effects of parent-child discrepancies on future child depressive symptoms may be that, in line with the *stage-environment fit theory* (Eccles et al., 1993), changes in which child reports become more negative and/or parent reports become more positive are normative. So, when children report less autonomy support over time relative to their parents this might be a sign of healthy adolescent functioning. Only in extreme cases, in which parent-child discrepancies change to a much higher extent than is normally expected within adolescence, this may be related to more child depressive symptoms than usual. As this was a relatively homogeneous, moderate-to-high SES sample, it is possible that such large within-family fluctuations in parent-child discrepancies were rare in this sample.

Prior researchers that found no evidence for within-family predictive effects argued that perhaps such causal processes played out in earlier life stages, before the observation window (Van Lissa & Keizer, 2020). The concurrent within-family and between-family patterns found between parent-child discrepancies and depressive symptoms may have stabilized before adolescence, and adolescent depressive symptoms could be the result of parent-child discrepancies during childhood or the other way around. Future research may be necessary to not only examine possible protective and risk factors in early family life that predict the development of negative perspectives on parental autonomy support, but also factors that predict the development of reporter differences between parents and children.

### Associations Between Parent-Child Discrepancies and Parent Depressive Symptoms

With regard to *parent* depressive symptoms, the findings showed no associations with parent-child discrepancies at the within- or between-family level. This contradicts prior work that found associations between mother depressive symptoms and reporter discrepancies, and attributed those effects to distorted perspectives of mothers with more depressive symptoms (e.g., Ehrlich et al., 2016). The present findings provide no evidence for the *depression-distortion hypothesis* but are in line with two previous studies that, like the current study, considered the mean report of the parent and the child and did not find associations between parent-child discrepancies and parents’ depressive symptoms (Korelitz, 2017; Ohannessian et al., 2016). In line with one of these studies (Ohannessian et al., 2016), the present findings suggest that only the mean report of parent and child is negatively associated with mothers’ depressive symptoms. By considering the average perspective, the current study provided a fuller picture of associations between perspectives on maternal autonomy support and mothers’ depressive symptoms, namely that only mean reports are related to depressive symptoms.

### Differences Between Father-Child and Mother-Child Discrepancies

In line with findings from previous studies (Ingoglia et al., 2021; Nelemans et al., 2016), the results demonstrate that, compared to father reports, mother reports were more positive relative to child reports. Nevertheless, both father-child and mother-child discrepancies did not predict future child depressive symptoms and there was no evidence that other associations between parent-child discrepancies and informants’ depressive symptoms differed between fathers and mothers. This is in contrast to a previous study on a Mexican American sample showing that only father-child discrepancies, and not mother-child discrepancies, in autonomy support were related to child adjustment (Sher-Censor et al., 2011). Although the researchers did not test the significance of this difference, they suggested that communication difficulties with regard to father autonomy support may be more strongly associated with child outcomes, because in their sample fathers were more likely to fulfill the authority figure role within the family. It might be the case that there were no differences between father-child and mother-child discrepancies in the association with depressive symptoms, because the current study is situated within a relatively higher educated sample of families in the Netherlands, in which family roles of fathers and mothers may be more similar due to egalitarian gender attitudes (McDaniel, 2008; Solera & Mencarini, 2018).

### Limitations and Future Directions

Although this research provided more insights into linkages between parent-child discrepancies and informants’ depressive symptoms, there are some limitations with regard to the latent congruence model that was applied. Compared to latent difference scores, polynomial regression might provide a more nuanced understanding of how different forms of congruence and incongruence relate to outcomes. By including linear and quadratic interaction terms this method tests whether the interaction between parent and child reports predicts the outcome variable over and above the main effects of individual reports. Significant interaction terms can be further investigated by post-hoc probing. This way, polynomial regression can more directly test whether informants’ depressive symptoms are higher when parents and children disagree about levels of autonomy support, agree about levels of autonomy support, or whether the effect of disagreement or agreement differs as a function of reported levels of autonomy support (e.g., when either informant reports lower levels of autonomy support). Nevertheless, the type of research questions that can be addressed with polynomial regression is rather limited and it is not possible to use parent-child discrepancies as predictor and outcome measures within the same model (de Haan et al., 2018). Since the main objective of the current study was to unravel the temporal ordering of parent-child discrepancies and informants’ depressive symptoms, latent difference scores were needed to assess parent-child discrepancies and to run a complex model differentiating within- from between-family associations.

Another limitation is that mechanisms linking parent-child discrepancies to informants’ depressive symptoms may operate on a different time scale. The significant concurrent within-family association between father-child discrepancies and child depressive symptoms could be the result of daily causal effects. When causal relations occur on a shorter (e.g., day-to-day) time level, yearly measurement waves may fail to capture over-time effects. Future studies are needed using different time intervals.

The generalizability of the findings is limited by the fact that the sample consisted predominantly of middle-to-high SES, two-parent families. There may be more agreement between children and their parents in such families (Hou et al., 2020), and parents from lower socioeconomic backgrounds may be more prone to socio-economic stress, which could influence the processes studied (Conger et al., 2010; Masarik & Conger, 2017). Future studies should include more diverse samples, which may show more variation in parent-child discrepancies and (parent) depressive symptoms.

## Conclusion

Despite prior evidence for associations between parent-child discrepancies in perceived autonomy support and depressive symptoms, little was known about the direction of effects. The present study addressed this knowledge gap by investigating longitudinal within-family linkages between informants’ depressive symptoms and parent-child discrepancies in autonomy support. The results provided no evidence for within-family predictive effects of parent-child discrepancies on future informants’ depressive symptoms or vice versa. The only within-family level association found was that, during periods in which child reports were more negative relative to father reports than usual, children reported higher levels of depressive symptoms than usual. Other associations were at the between-family level: in families where child reports were more negative relative to parent reports, children reported higher levels of depressive symptoms compared to other families, and these associations were found over and above the associations with level of autonomy support. There were no associations between parents’ depressive symptoms and parent-child discrepancies. The findings suggest that associations between parent-child discrepancies and child depressive symptoms in adolescence mainly reflect stable differences between families rather than causal changes over time. No support was found for the notion that parent-child discrepancies in adolescence are a risk factor for, or a consequence of, informants’ depressive symptoms.

## Supplementary Information

Supplementary Information
