# Supplementary material for: Longitudinal Linkages Between Parent-Child Discrepancies in Reports on Parental Autonomy Support and Informants’ Depressive Symptoms
Source: J Youth Adolesc. 2023 Jan 24;52(4):899–912. doi: 10.1007/s10964-022-01733-y (PMC9957896; doi:10.1007/s10964-022-01733-y)
Supplement: Supplementary file 1 — Supplementary Information [file 10964_2022_1733_MOESM1_ESM.docx]

**Appendix A**

*Cronbach’s Alphas of Observed Variables per Wave*

| Wave | 1 | 2 | 3 | 4 | 5 | 6 |
| --- | --- | --- | --- | --- | --- | --- |
|  |  |  |  |  |  |  |
| **Autonomy support father** |  |  |  |  |  |  |
| Child-reported | .79 | .84 | .86 | .84 | .88 | .89 |
| Father-reported | .84 | .85 | .86 | .87 | .85 | .89 |
| **Autonomy support mother** |  |  |  |  |  |  |
| Child-reported | .85 | .86 | .86 | .86 | .87 | .89 |
| Mother-reported | .85 | .87 | .86 | .89 | .89 | .90 |
|  |  |  |  |  |  |  |
| **Depressive symptoms** |  |  |  |  |  |  |
| Child-reported | .93 | .94 | .94 | .95 | .94 | .94 |
| Father-reported | .81 | .87 | .88 | .87 | .91 | .91 |
| Mother-reported | .90 | .88 | .90 | .88 | .89 | .88 |

**Appendix B**

*Simplified Representation of RI-CLPM*


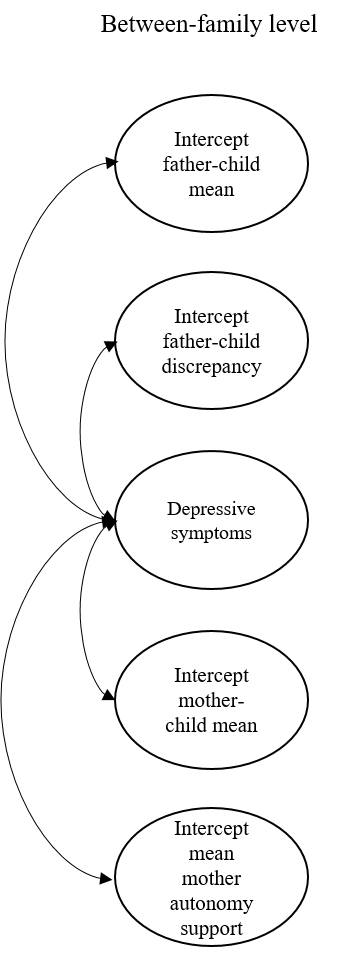

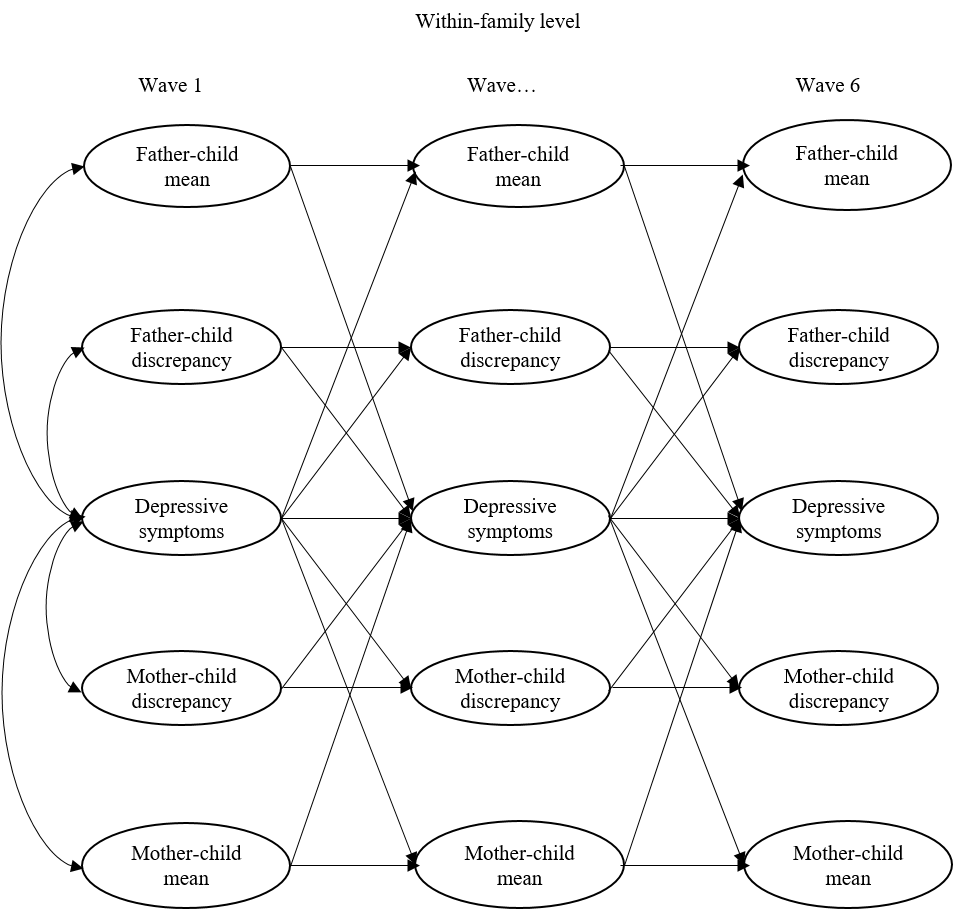


*Note.* Simplified representation of RI-CLPM, all possible between- and within-family associations are modelled, but not visualized. Depressive symptoms of child, father and mother are individually included in the model, but not visualized. Parent-child mean = latent variable of the average scores of parent and child report on autonomy support, parent-child discrepancy = latent difference score of parent and child report on autonomy support

**Appendix C**

*Concurrent Correlations per Wave*

| Wave 1 | 1 | 2 | 3 | 4 | 5 | 6 |
| --- | --- | --- | --- | --- | --- | --- |
| 1. Child-reported father support |  |  |  |  |  |  |
| 2.Child-reported mother support | 0.64*** |  |  |  |  |  |
| 3.Father-reported support | 0.13** | 0.01 |  |  |  |  |
| 4.Mother-reported support | 0.21*** | 0.18*** | 0.19*** |  |  |  |
| 5.Child depressive symptoms | -0.34*** | -0.23*** | -0.12* | -0.11* |  |  |
| 6.Father depressive symptoms | -0.03 | 0.00 | 0.06 | 0.04 | 0.06 |  |
| 7.Mother depressive symptoms | -0.09* | -0.07 | -0.04 | -0.03 | 0.21*** | 0.09 |

| Wave 2 | 1 | 2 | 3 | 4 | 5 | 6 |
| --- | --- | --- | --- | --- | --- | --- |
| 1. Child-reported father support |  |  |  |  |  |  |
| 2.Child-reported mother support | 0.61*** |  |  |  |  |  |
| 3.Father-reported support | 0.22*** | 0.18*** |  |  |  |  |
| 4.Mother-reported support | 0.18*** | 0.22*** | 0.24*** |  |  |  |
| 5.Child depressive symptoms | -0.28*** | -0.19*** | -0.12* | -0.11* |  |  |
| 6.Father depressive symptoms | -0.01 | 0.01 | -0.02 | 0.12* | -0.02 |  |
| 7.Mother depressive symptoms | -0.08 | -0.08 | -0.05 | -0.03 | 0.23*** | 0.21*** |

| Wave 3 | 1 | 2 | 3 | 4 | 5 | 6 |
| --- | --- | --- | --- | --- | --- | --- |
| 1. Child-reported father support |  |  |  |  |  |  |
| 2.Child-reported mother support | 0.57*** |  |  |  |  |  |
| 3.Father-reported support | 0.26*** | 0.19*** |  |  |  |  |
| 4.Mother-reported support | 0.18*** | 0.14** | 0.22*** |  |  |  |
| 5.Child depressive symptoms | -0.28*** | -0.23*** | -0.14** | -0.05 |  |  |
| 6.Father depressive symptoms | 0.07 | 0.08 | 0.03 | 0.04 | 0.12 |  |
| 7.Mother depressive symptoms | -0.06 | -0.04 | 0.00 | 0.01 | 0.22*** | 0.23*** |

| Wave 5 | 1 | 2 | 3 | 4 | 5 | 6 |
| --- | --- | --- | --- | --- | --- | --- |
| 1. Child-reported father support |  |  |  |  |  |  |
| 2.Child-reported mother support | 0.58*** |  |  |  |  |  |
| 3.Father-reported support | 0.26*** | 0.21*** |  |  |  |  |
| 4.Mother-reported support | 0.18*** | 0.12* | 0.25*** |  |  |  |
| 5.Child depressive symptoms | -0.22*** | -0.27*** | -0.07 | -0.05 |  |  |
| 6.Father depressive symptoms | 0.06 | 0.07 | 0.00 | 0.00 | 0.07 |  |
| 7.Mother depressive symptoms | 0.01 | -0.05 | -0.04 | -0.13** | 0.25*** | 0.23*** |

| Wave 4 | 1 | 2 | 3 | 4 | 5 | 6 |
| --- | --- | --- | --- | --- | --- | --- |
| 1. Child-reported father support |  |  |  |  |  |  |
| 2.Child-reported mother support | 0.66*** |  |  |  |  |  |
| 3.Father-reported support | 0.18*** | 0.14** |  |  |  |  |
| 4.Mother-reported support | 0.21*** | 0.17*** | 0.22*** |  |  |  |
| 5.Child depressive symptoms | -0.29*** | -0.24*** | -0.13* | -0.10* |  |  |
| 6.Father depressive symptoms | 0.11* | 0.11* | 0.03 | 0.04 | 0.04 |  |
| 7.Mother depressive symptoms | -0.06 | -0.13** | 0.00 | -0.09 | 0.21*** | 0.12* |

| Wave 6 | 1 | 2 | 3 | 4 | 5 | 6 |
| --- | --- | --- | --- | --- | --- | --- |
| 1. Child-reported father support |  |  |  |  |  |  |
| 2.Child-reported mother support | 0.52*** |  |  |  |  |  |
| 3.Father-reported support | 0.23*** | 0.19*** |  |  |  |  |
| 4.Mother-reported support | 0.13** | 0.22*** | 0.16** |  |  |  |
| 5.Child depressive symptoms | -0.29*** | -0.21*** | -0.10* | -0.01 |  |  |
| 6.Father depressive symptoms | 0.01 | 0.07 | 0.01 | -0.01 | 0.14 |  |
| 7.Mother depressive symptoms | -0.13** | -0.07 | -0.05 | -0.04 | 0.20*** | 0.21*** |

*Note.* Support = autonomy support, * *p* < .05, ** *p* < .01, *** *p* <.001
